# Transthyretin Cardiac Amyloidosis in an Elderly Male With Heart Failure Intolerant to Guideline-Directed Medical Therapy

**DOI:** 10.7759/cureus.62722

**Published:** 2024-06-19

**Authors:** Noah Ene, Toyin Ingram, Manoj Bhandari

**Affiliations:** 1 Internal Medicine, Cape Fear Valley Health, Fayetteville, USA; 2 Cardiology, Cape Fear Valley Medical Center, Fayetteville, USA

**Keywords:** wild-type attr amyloidosis, hereditary attr amyloidosis, serum electrophoresis, guideline-directed medical therapy (gdmt), technetium pyrophosphate scintigraphy, conduction abnormality, heart failure, transthyretin amyloid, light chain amyloid, cardiac amyloidosis

## Abstract

Cardiac amyloidosis arises when there is a deposition of abnormal proteins, called amyloids, in the myocardium. It can manifest as overt heart failure, conduction abnormalities, atrial and ventricular arrhythmia, cardiomyopathy, and aortic stenosis. Two main types of proteins identified in cardiac amyloidosis are light-chain amyloid and transthyretin amyloid. Cardiac amyloidosis, although common, is an underdiagnosed cause of heart failure in many cases. A high index of suspicion is needed to make a diagnosis, given that symptoms are not specific. Early diagnosis and treatment of cardiac amyloidosis are associated with reduced morbidity and improved survival. We present a case of a 73-year-old African American male with decompensated heart failure with reduced ejection fraction intolerant to guideline-directed medical therapy who was later found to have cardiac amyloidosis.

## Introduction

Cardiac amyloidosis arises when there is a deposition of abnormal proteins, called amyloids, in the myocardium. It can manifest as overt heart failure, conduction abnormalities, atrial and ventricular arrhythmia, cardiomyopathy, and aortic stenosis [1,2]. Cardiac amyloidosis can occur in isolation or as part of amyloidosis with systemic multiorgan involvement. The prognosis is poor with cardiac involvement. Two main types of proteins identified in cardiac amyloidosis are light-chain amyloid (AL) and transthyretin amyloid (ATTR) [1,3]. ATTR consists of hereditary and acquired or wild types. The first line for diagnosis is a cardiac ultrasound that may demonstrate bi-atrial and bi-ventricular wall thickening and septal thickening, and strain imaging reveals impaired longitudinal strain in mid-ventricular and basal segments with sparing of the apical segment. Cardiac MRI is used to complement cardiac ultrasound and helps differentiate cardiac amyloidosis from other causes of hypertrophic cardiomyopathy by demonstrating late gadolinium enhancement. An MRI is also needed to assess the response to therapy [4]. An EKG shows a low voltage. When cardiac amyloidosis is suspected or diagnosed, a test for serum-free light chains and serum and urine immunofixation is done to check for AL type. Technetium-pyrophosphate scintigraphy is needed for the diagnosis of ATTR. This is followed by genetic counseling and testing to determine if it’s acquired ATTR (ATTRwt) or hereditary ATTR (ATTRv) [5]. Traditional therapies for the management of heart failure are usually not indicated due to side effects [2]. The development of novel therapies has led to improved outcomes in cardiac amyloidosis when administered early in the course of the disease [2,6]. We present the case of an elderly African American male with heart failure who was found to have ATTR.

## Case presentation

A 73-year-old African American male with a history of heart failure with reduced ejection fraction (HFrEF), chronic obstructive pulmonary disease on 2 L oxygen at home, hypertension, and coronary artery disease status post coronary artery bypass graft was seen for management of heart failure exacerbation following presentation with worsening shortness of breath at rest, orthopnea, and bilateral lower extremity swelling for a month. Results of a transthoracic echocardiogram one month prior noted severely reduced left ventricular systolic function with ejection fraction (EF) estimated at 25-30% and moderately reduced right ventricular systolic function. He was intolerant of guideline-directed medical therapy (GDMT) for heart failure due to hypotension and hence required blood pressure support with midodrine. He was unable to ambulate in the previous six months.

In the ED, his vital signs were as follows: blood pressure 113/91 mmHg, heart rate 123 bpm, respiratory rate 27 breaths/min, temperature 97.8 Fahrenheit (36.5 Celsius), and oxygen saturation 95% while on 10 L oxygen via a non-rebreather mask. Physical examination was significant for respiratory distress, S3, bibasilar rales in the lungs, ascites, and 3+ bilateral pedal pitting edema. Laboratory results are listed in Table 1.

**Table 1:** Laboratory test results BUN: blood urea nitrogen, AST: aspartate transaminase, ALT: alanine transaminase, eGFR: estimated glomerular filtration rate, pCO2: partial pressure of carbon dioxide, HCO3: bicarbonate

Parameters	Patient’s values	Reference ranges
Sodium	139 mmol/L	136-145 mmol/L
Potassium	4.6 mmol/L	3.4-4.9 mmol/L
BUN	54 mg/dL	7-25 mg/dL
Creatinine	2.09 mg/dL	0.6-1.3 mg/dL
Glucose	121 mg/dL	74-109 mg/dL
Calcium	9.3 mg/dL	8.6-10.2 mg/dL
Albumin	3.1 g/dL	3.5-5.7 g/dL
AST	77 U/L	13-39 U/L
ALT	49 U/L	7-52 U/L
Alkaline phosphatase	372 U/L	30-105 U/L
Bilirubin total	1.9 mg/dL	0.3-1 mg/dL
Total protein	7.7 g/dL	6.4-8.9 g/dL
eGFR	33 mL/min/1.73m*2	>60 mL/min/1.73m*2
White cell count	6900 c/uL	4500-12500 c/uL
Hemoglobin	10.8 g/dL	13.5-18 g/dL
Platelets	186000 c/uL	150000-450000 c/uL
pH	7.31	7.32-7.43
pCO2	65 mmHg	41-51 mmHg
HCO3	32.7 mmHg	22-26 mmHg
B-type natriuretic peptide	1994 pg/mL	<100 pg/mL
Maximum high-sensitivity troponin	398 pg/mL	2-20 pg/mL
Urine protein/creatinine	547.05 mg/g	<200 mg/g

**Figure 1:**
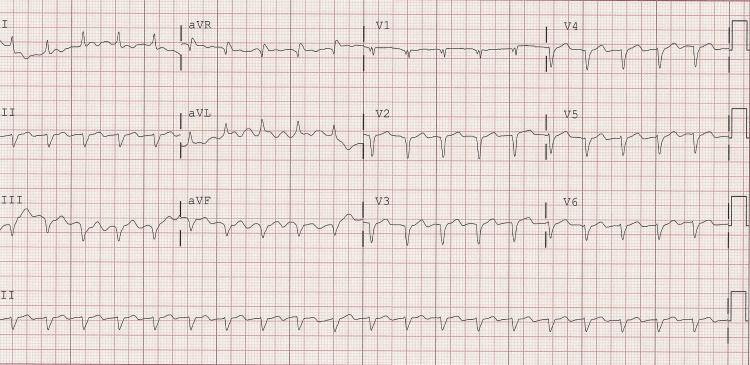
EKG with low voltage, intraventricular conduction delay, Q waves in anterior, and inferior and lateral leads EKG: electrocardiogram

EKGs noted sinus tachycardia at 122 bpm, low voltages, nonspecific intraventricular conduction delay, left axis deviation, normal intervals, and old anterior and inferior-lateral infarcts, as shown in Figure 1.

**Figure 2:**
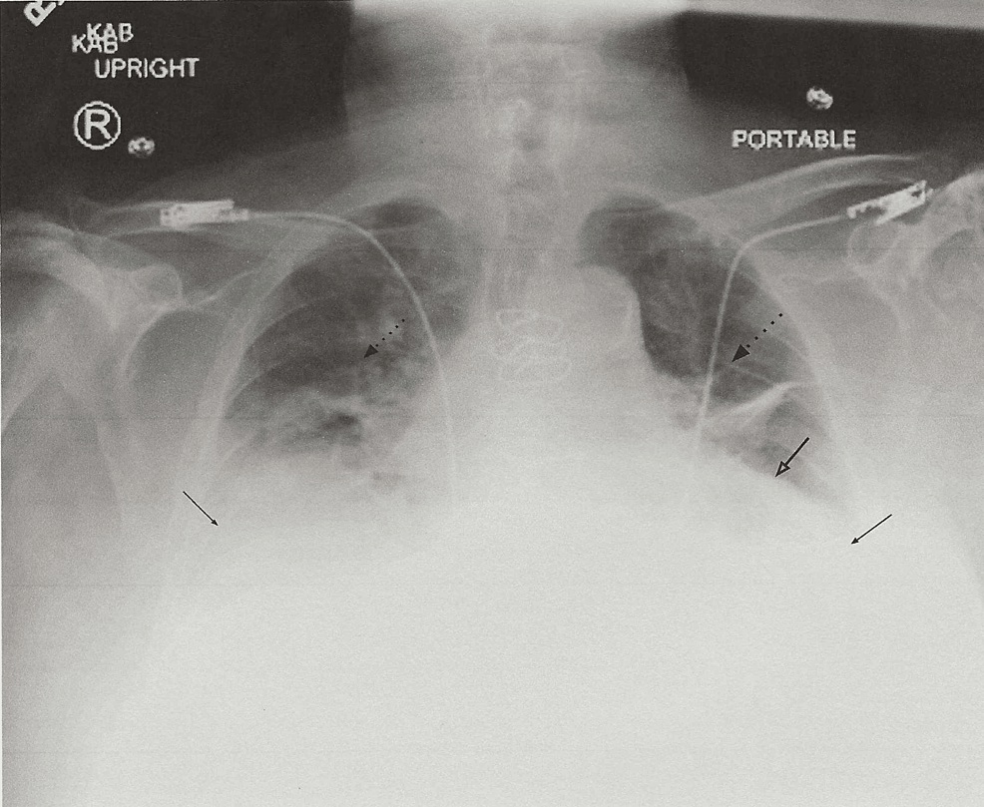
Chest X-ray showing cardiomegaly (arrow with open head), interstitial opacities (broken arrow), and bilateral pleural effusions (solid arrow with closed head)

Chest X-ray revealed increased bilateral interstitial opacities, prominent pulmonary vessels, cardiomegaly, and bilateral pleural effusions (Figure 2). A ventilation-perfusion scan was done with a low probability of acute PE. He was initiated on diuretic therapy with intravenous bumetanide.

**Figure 3:**
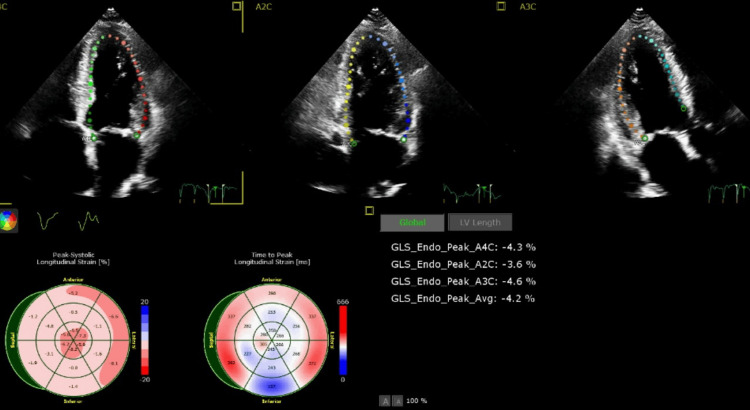
Limited 2D echocardiogram showing decreased global longitudinal strain with relatively better strain (intensely pink area) at the apex compared to other segments 2D: two-dimensional

During hospitalization, he developed atrial tachycardia that was managed with amiodarone. He responded adequately to diuresis with improvement in respiratory status back to baseline oxygen requirement of 2 L/min. The addition of metoprolol succinate and empagliflozin was met with recurrent hypotension and worsening renal function, necessitating discontinuation of therapy. A limited transthoracic echocardiogram with strain noted EF 30-35%, severe global hypokinesis, and decreased global longitudinal strain with relatively better strain at the apex compared to other segments with concern for amyloidosis (Figure 3).

Serum protein electrophoresis revealed elevated free kappa and lambda light chains at 142.2 mg/L and 99.9 mg/L, respectively, and the kappa/lambda ratio was normal at 1.42 (Table 2).

**Table 2:** Serum protein electrophoresis

Parameters	Patient’s values	Reference ranges
Immunoglobulin G	2413 mg/dL	603-1613 mg/dL
Immunoglobulin A	422 mg/dL	61-437 mg/dL
Immunoglobulin M	50 mg/dL	15-143 mg/dL
Monoclonal spike	Not observed	Not observed
Free kappa Lt chains	142.2	3.3-19.4 mg/L
Free lambda Lt hains	99.9 mg/L	5.7-26.3 mg/L
Kappa/Lambda ratio, S	1.42	0.26-1.65

**Figure 4:**
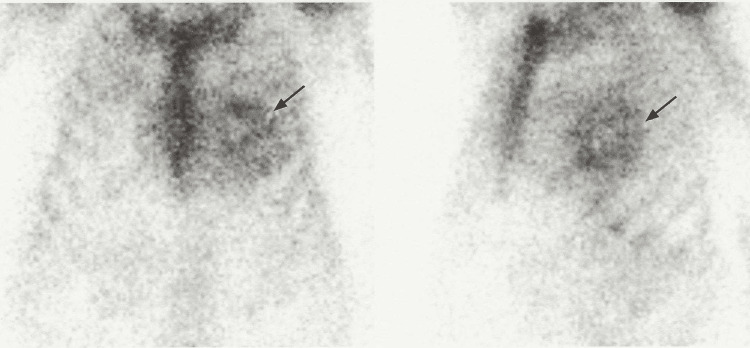
Technetium pyrophosphate scintigraphy with increased uptake of radiotracer (black arrow) within the heart and similar to bone (Grade 2)

Nuclear medicine cardiac amyloidosis scan (technetium pyrophosphate scintigraphy) was strongly suggestive of ATTR with a semi-quantitative visual score of 2 and H/CL ratio of 1.84, as shown in Figure 4.

Right heart catheterization was done with the following findings: mean pulmonary artery pressure 32 mmHg, pulmonary capillary wedge pressure 22 mmHg, diastolic pressure gradient <7 mmHg, cardiac output 4.13 L/min, cardiac index 1.84 L/min/m2, and pulmonary vascular resistance 2.18 WU, suggesting isolated post-capillary pulmonary hypertension.

Goals of care were discussed given the overall poor prognosis, with the family declining further therapy. GDMT was discontinued, and he was discharged on midodrine for blood pressure support and amiodarone with a plan to follow outpatient cardiology. Two months later, he was hospitalized for treatment of severe pneumonia. The patient was placed in comfort care and passed away after six weeks of hospitalization.

## Discussion

Cardiac amyloidosis is a form of infiltrative-restrictive cardiomyopathy. It is caused by the deposition of abnormally misfolded proteins called amyloids in the myocardium [1]. There are two main types of cardiac amyloidosis: AL amyloidosis, which is more common, and ATTR, which can be ATTRv or ATTRwt [1,3]. AL amyloidosis occurs due to increased production of abnormal kappa and lambda chains from the bone marrow and is seen in plasma cell dyscrasias. Transthyretin is a protein that is produced by the liver and is needed for the transport of retinol and thyroxine. A mutation in the gene coding for this protein will lead to the formation of abnormal transthyretin proteins that tend to misfold easily and deposit in tissues, as is the case with ATTRv. Common mutations include Val122ile (V122), Val30Met (V30M), and Thr60Ala (T60A) [1,3]. ATTRwt usually occurs in men over the age of 60 and involves normal transthyretin protein with an increased tendency to misfold and deposit in tissues as one gets older [1]. Although uncommon, secondary amyloidosis due to serum amyloid A protein can in some cases affect the heart; this is usually seen in poorly controlled chronic inflammatory conditions [1]. Cardiac amyloidosis can manifest as overt heart failure, conduction abnormalities, atrial and ventricular arrhythmia, cardiomyopathy, and aortic stenosis [1,2,4].

The true prevalence of cardiac amyloidosis remains unknown. A cardiac amyloid post-mortem study found AL amyloidosis in 54% of cases and ATTR in 42% of cases, and the remainder was unclassified [4]. The epidemiology of the disease, classically considered rare and incurable, has radically changed in the last few years due to significant advances in diagnostic and therapeutic strategies, arousing growing interest in the scientific community [7]. Cardiac amyloidosis is considered a rare disease occurring in fewer than five people in 10,000; epidemiological data is mostly derived from single-center studies or population registries [8,9]. As such, a high index of suspicion is needed to make a diagnosis of cardiac amyloidosis. In our case, cardiac amyloidosis was suspected given that the patient was no longer tolerant of GDMT for heart failure, prompting further investigation.

Typically, patients with cardiac amyloidosis will have an EKG that classically shows disproportionately low voltage QRS with relatively increased left ventricular thickness seen in echocardiograms and pseudo-Q waves. Whenever hypertrophic cardiomyopathy or hypertensive cardiomyopathy is considered, it is prudent to also include cardiac amyloidosis as a differential until proven otherwise. Hints to delineate the above differentials include findings from echocardiograms that point to cardiac amyloidosis, which include abnormal longitudinal strain at the base but normal strain at the apex, also known as apical sparing or cherry on top pattern [4-6]. This finding is highly specific for differentiating cardiac amyloidosis from hypertrophic cardiomyopathy and hypertensive cardiomyopathy [5]. Once these suspicions are found on the EKG and echocardiogram, diagnosis requires further investigation to distinguish between AL amyloidosis and ATTR using serum and urine electrophoresis with immunofixation and a technetium pyrophosphate bone scan. If both tests are positive, the diagnosis is generally AL amyloidosis or may be ATTR with monoclonal gammopathy of undetermined significance. A biopsy is only indicated to establish amyloidosis and distinguish AL amyloidosis from ATTR in patients who have abnormal monoclonal protein testing and positive scintigraphy. Biopsies can be obtained from the periumbilical fat pad, bone, or endomyocardium. An endomyocardial biopsy should only be done if samples from the periumbilical fat pad and bone are negative for amyloid deposits. Other supportive tests include cardiac MRI and genetic testing. Genetic testing is useful to distinguish between ATTRv and ATTRwt [4,5]. This is important given that if a patient has ATTRv, the children will benefit from genetic screening.

The management of cardiac amyloidosis is dependent on the type. AL amyloidosis is managed with chemotherapy, immunotherapy, and autologous hematopoietic cell transplantation. ATTR can be managed with transthyretin stabilizers such as tafamidis and diflunisal, transthyretin silencers such as patisiran and inotersen used in ATTRv, and transthyretin disruptors such as doxycycline, tauroursodeoxycholic acid, and nutraceuticals [1,4,6]. Definitive treatment involves cardiac transplants in AL amyloidosis and both cardiac and liver transplants in ATTR [1]. The average survival time after diagnosis for cardiac amyloidosis is as follows: six months for AL amyloidosis, 2.6 years for ATTRv, and 3.6 years for ATTRwt [4].

Management of HFrEF in patients with cardiac amyloidosis is difficult given the pathophysiological effect of cardiac amyloidosis on HFrEF. Due to left ventricular stiffness, the cavity is unable to expand to receive increased venous return, leading to increased preload reserve. This in turn causes pulmonary capillary wedge pressure to increase despite reduced preload, which precludes the use of substantial doses of diuretics. Angiotensin-converting enzyme inhibitors, which have a vasodilatory effect, may lead to profound hypotension due to severely limited stroke volume reserve and autonomic dysfunction. This leads to the dependence of cardiac output on heart rate, causing resistance to beta blockers. Of note, digoxin should also be avoided, as it can cause high toxicity in patients with ATTR since it binds to amyloid [1]. As seen in our patient, he developed persistent hypotension with the addition of metoprolol succinate, requiring therapy with midodrine. He also had atrial tachycardia that was responsive to amiodarone.

The true prevalence of cardiac amyloidosis remains unknown. More cases of cardiac amyloidosis are being discovered, given advancements in diagnostic tools. Cardiac amyloidosis carries a poor prognosis. Therefore, it is paramount for clinicians to have a high index of suspicion to diagnose cardiac amyloidosis, given that there are novel therapies that have been shown to improve survival and slow down disease progression. This case report adds to the number of amyloidosis cases diagnosed and also highlights the importance of working up suspected patients for amyloidosis, including those with an established diagnosis of heart failure.

## Conclusions

Cardiac amyloidosis presents with non-specific symptoms, including conduction abnormalities, arrhythmias, and heart failure. In our case, cardiac amyloidosis was suspected given the new intolerance to GDMT for heart failure. Limited cardiac 2D echocardiography with straining revealed decreased longitudinal strain with apical sparing typical for cardiac amyloidosis, which could be AL amyloidosis or ATTR. Serum protein electrophoresis makes AL amyloidosis less likely. The technetium pyrophosphate scan was positive for ATTR, which could be ATTRv or ATTRwt. Genetic testing was not completed in this case, per the family’s preference. This case highlights the importance of having a high index of suspicion in order to make a diagnosis of cardiac amyloidosis, given that the patient could no longer tolerate GDMT in the absence of any significant change in left ventricular dysfunction. The overall prognosis of cardiac amyloidosis has improved with the administration of appropriate therapy early in the disease course.
